# Fixed- versus mobile-bearing unicompartmental knee arthroplasty: a meta-analysis

**DOI:** 10.1038/s41598-020-76124-z

**Published:** 2020-11-05

**Authors:** Wenchao Zhang, Jianpeng Wang, Hui Li, Wanchun Wang, Daniel M. George, Tianlong Huang

**Affiliations:** 1 Department of Orthopaedics, The Second Xiangya Hospital, Central South University, No.139 Middle Renmin Road, Changsha, Hunan 410011 People’s Republic of China; 2 Department of Orthopaedics, Honghui Hospital, Xi’An Jiaotong University, Xi’an, Shanxi People’s Republic of China; 3 Department of Orthopaedics, Royal Adelaide Hospital, Adelaide, Australia

**Keywords:** Anatomy, Outcomes research, Trauma

## Abstract

Unicompartmental knee arthroplasty (UKA) can be either a fixed bearing (FB) or a mobile bearing (MB) construct with controversy as to which design is superior. This question is addressed with a systematic review and meta-analysis. A literature search was performed using PubMed, Embase and the Cochrane Library. Studies were reviewed according to the inclusion and exclusion criteria developed in advance. We compared the differences in clinical and radiological outcomes between the FB and MB UKAs. Analyses were performed with the Review Manager and STATA software. A total of 17 studies involving 2612 knees were included. No significant differences were presented between the FB and MB prostheses in clinical and radiological outcomes. However, it was evident that there were differences in the modes and timing of the failures, bearing dislocation led to earlier failures in the MB prosthesis, while the FB prosthesis failed later due to polyethylene wear. There was no evidence of publication bias using the incidence of revisions. There is no significant difference between the FB and MB UKAs; however, there are differences in the modes and timing of failures.

## Introduction

UKA was originally introduced as a FB construct to address unicompartmental osteoarthritis of the knee^1^. More recently MB constructs were introduced to minimize contact stress and polyethylene wear^2,3^. Each prosthesis design has its own strengths and weaknesses and there remains discussion on which prosthetic design has superior long-term results. The MB prosthesis, by offering more congruent bearing surfaces with a large contact area generates less contact stresses, theoretically decreasing the risk of aseptic loosening, polyethylene wear, and implant revision in the long term^4^. However, the MB prosthesis is technically more difficult to implant. Without precise alignment and ligament balancing, it can lead to bearing dislocation or impingement causing increased wear^5^. Conversely, the FB prosthesis often has a flat tibial articular surface, which is technically easier to implant and there is no risk of bearing dislocation^1,6^. However, the advantages are also the disadvantages, the FB prosthesis is less conforming as flexion occurs and can lead to point loading due to the flat tibial articular surface^7^. Direct comparison between FB and MB prostheses have been made in several studies, however, findings were inconsistent and unable to demonstrate an advantage of one prosthesis design over the other^5,8,9^. A previous meta-analysis attempted to resolve this debate but was limited by a lack of clinical studies to support a robust statistical analysis^2^. However, since then further studies have been published, which allows a more in-depth analysis. Some recent studies have demonstrated higher knee scores and lower revision rates of the MB prosthesis compared to the FB prosthesis^10–12^, while other studies have demonstrated the opposite relationship^13–15^. In addition, previous retrospective cohort studies comparing mid-term and long-term survivorship between the two UKA prostheses did not demonstrate obvious differences^1,10,16^.

In this meta-analysis we evaluate the performance of FB and MB prostheses comparing clinical and radiological outcomes as well as complications and reported survivorship.

## Results

**Figure 1:**
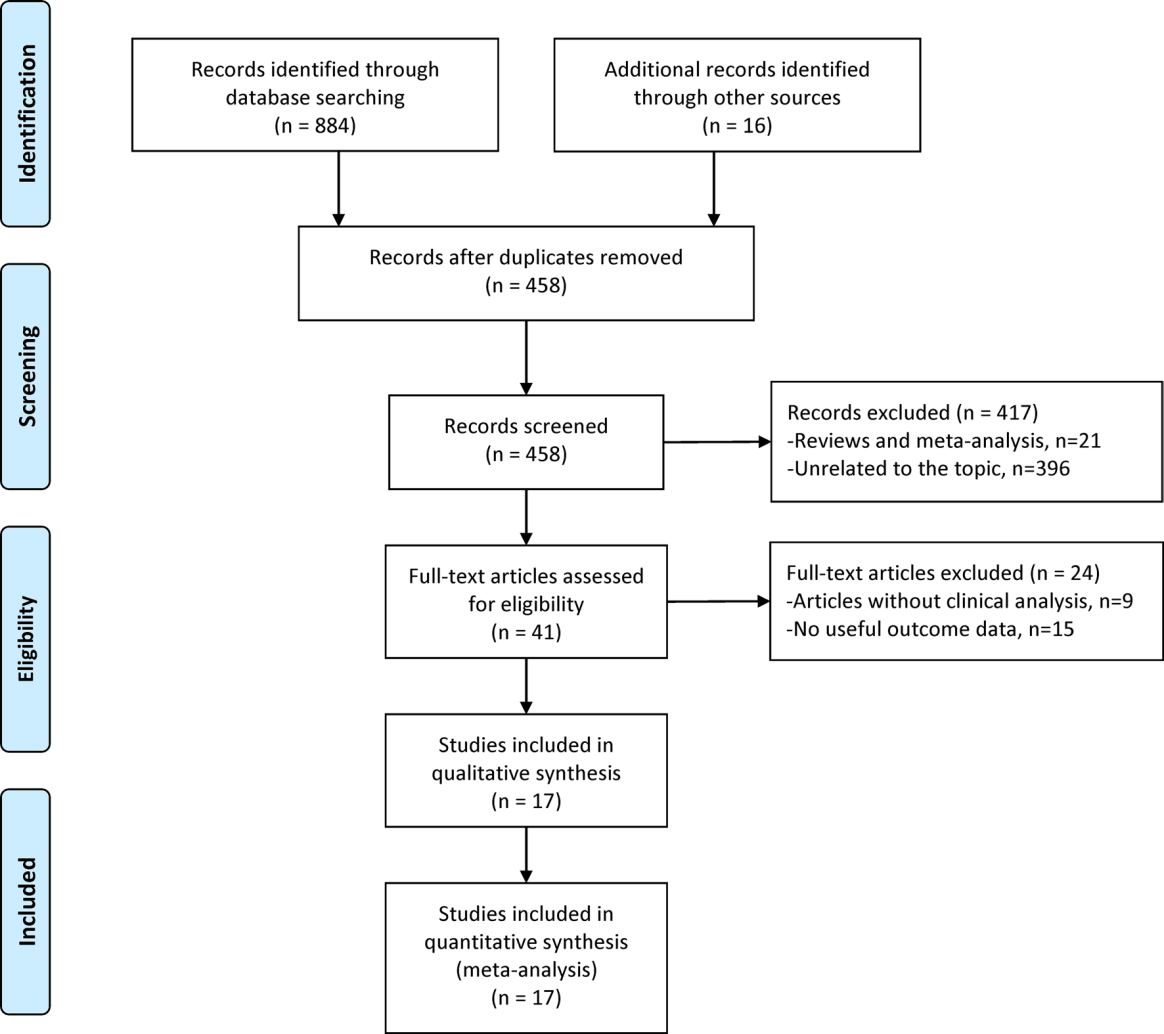
Flowchart for the identification of eligible studies. This flowchart covered the detailed selecting process, including three databases and relevant studies, the excluded reasons and number of studies, and the final studies included in the meta-analysis.

A total of 900 studies were identified in this study. After review of the titles, abstracts, full articles, and excluding unrelated articles 17 studies involving 2612 knees were eligible and selected for the final meta-analysis^1,5,8–22^ (Fig. 1). Three of the 17 eligible studies were randomized controlled trials (RCTs). Weight or body mass index (BMI) was reported in 11 studies. The mean follow-up period ranged from 7-months to 17.2-years; the follow-up period was not stated in two studies. Additional details about study characteristics and participant demographics are shown in Table 1.

**Table 1:** Study characteristics and participant demographics.

Author	Type of study	Technique	Number of knees (patients)	Mean age (year)	BMI/weight (Kg)	Follow-up (year)	Level of evidence
Bhattacharya^13^	Retrospective	FB	91 (79)	67.7	NA	3.7	III
		MB	49 (44)	68.8		5.6	
Biau^17^	Retrospective	FB	67 (57)	66	28	3.25	III
		MB	37 (33)	60	32	5.25	
Bini^14^	Registry	FB	632	NA	NA	NA	II
		MB	350				
Catani^8^	Retrospective	FB	10 (10)	70.3 (7.6)	76.3 (14.4)	1.03 (0.6)	III
		MB	10 (10)	70.3 (5.8)	75.4 (11.9)	3.84 (2.07)	
Confalonieri^9^	RCT	FB	20 (20)	69.5	NA	5.7	I
		MB	20 (20)	71		5.7	
Emerson^1^	Retrospective	FB	51 (45)	63	84.4	6.1	III
		MB	50 (43)	63	79.4	6.8	
Emerson^21^	Retrospective	FB	42 (39)	NA	NA	NA	III
		MB	27 (21)				
Gleeson^5^	RCT	FB	57 (49)	66.7	83	4	I
		MB	47 (43)	64.7	77.7	4	
Inoue^12^	Retrospective	FB	24	75 (6.5)	23.7 (6.1)	38.2 months	III
		MB	28	73.3 (7.5)	25.2 (3.4)	27.3 months	
Li^20^	RCT	FB	28 (24)	70	26.5	2	I
		MB	28 (24)	74	27.6	2	
Lu^19^	Retrospective	FB	45 (45)	67.5 (9.5)	23.36 (3.53)/64.3 (14)	7–14 months	III
		MB	45 (45)	65.5 (13.5)	22.88 (3.56)/64.8 (12.80)	7–14 months	
Neufeld^18^	Retrospective	FB	68 (56)	64.6	29.7	10	III
		MB	38 (33)	60.3	32.7	10	
Ozcan^11^	Retrospective	FB	153	NA	NA	28.8 (9.7) months	III
		MB	171			31 (11.3) months	
Parratte^10^	Retrospective	FB	79 (75)	62.8 (9.2)	26 (4)	17.2 (4.8)	III
		MB	77 (72)	63.4 (11)	27 (3)	17.2 (4.8)	
Verdini^15^	Observationa	FB	8 (8)	67	28	20 months	III
		MB	7 (7)	68	28	23 months	
Tecame^22^	Retrospective	FB	15 (15)	48.4	NA	42 (6.7) months	III
		MB	9 (9)	47.8		53 (8.3) months	
Whittaker^16^	Retrospective	FB	150 (117)	68	28.7	8.1	III
		MB	79 (62)	63	30.7	3.9	

*BMI* body mass index, *FB* fixed-bearing, *MB* mobile-bearing, *RCT* randomized controlled trial, *y* years, *NA* not applicable.

Within the 17 eligible studies the FB knee prostheses were from seven manufacturers: Miller-Galante (Zimmer), Robert Brigham (Johnson and Johnson), St Georg Sled (Waldemar Link), Preservation All Poly (DePuy), Optetrak (Exactech), Allegreto (Centerpulse), and Accuris (Smith and Nephew); the MB knee prostheses were from two manufacturers: Oxford (Biomet) and AMC (Alphanorm).

### Clinical outcomes

**Figure 2:**
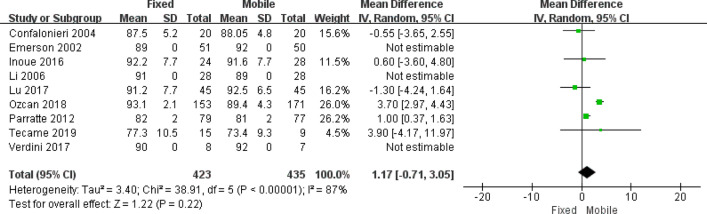
Forest plot for the Knee society score following the FB and MB UKAs. Nine studies were analyzed, while only six studies were showed in the forest plot for absence of standard deviation (SD) in the other three studies. Since I^2^ = 87%, a random effect model was used (RevMan 5, version 5.3, URL: https://training.cochrane.org).

Nine studies assessed the Knee Society Score (KSS)^1,9–12,15,19,20,22^; six studies assessed the Western Ontario and McMaster Universities Osteoarthritis Index (WOMAC) score^11,16–18,20,22^; three studies assessed the Oxford Knee Score (OKS)^5,15,17^; one study assessed the Italian Orthopedic UKR’s Users Group (GIUM) score^9^; one study assessed the Bristol knee score^5^; and one study assessed the International Knee Society score^8^. No significant statistical differences were reported between the FB and MB groups on all knee scores measured in these studies. Although statistical heterogeneity was high in the assessment of the Knee Society Score, data from the meta-analysis did not demonstrate significant statistical differences between the FB and MB groups (*p* = 0.22; I^2^ = 87%) (Fig. 2). Two studies^1,20^ found similar results between FB and MB groups in respect to pain (*p* > 0.05), however, Gleeson et al.^5^ reported that the total pain score, as assessed using the Bristol Knee Score, was significantly greater in the FB group at 2 years (*p* = 0.013). Postoperative ROM was assessed in five studies^9,12,15,19,20^, no significant difference was shown between the FB and MB groups (*p* = 0.19; WMD = − 1.53; 95% CI − 3.80 to 0.74).

### Radiological outcomes

According to the Kennedy and White classification, which considers correct alignment when the mechanical axis is in Zone 2 or C (central) and malalignment in Zone 1 or Zone 3^23^. Malalignment occurred in 16 (8.0%) knees in the FB group and 22 (11%) knees in the MB group (OR = 0.70; 95% CI 0.36–1.38; *p* = 0.31). Two studies^12,19^ reported postoperative FTA and 3 studies^10,19,20^ reported postoperative HKA, no significant differences were found (*p* = 0.1; WMD = 0.60; 95% CI − 0.11 to 1.31 and *p* = 0.69; WMD = − 0.51; 95% CI − 3.03 to 2.01) between the FB and MB groups.

The incidence of radiolucent lines at the bone-tibial implant interface was examined in four studies. Li et al.^20^ reported a greater incidence of radiolucent lines at 2-years postoperatively in the FB group (37% vs. 8%; *p* = 0.02), while others demonstrated an increased incidence of radiolucent lines in the MB group (10.3% vs. 23.5%, *p* = 0.229; 69% vs. 24%, *p* = 0.001)^10,18^. Meanwhile, Andrea et al.^22^ revealed that no differences were found in RLLs between FM and MB group. Based on the cumulative data, no significant difference was found in the frequency of RLLs between the FB and MB groups (42.0% vs. 19.0%, OR = 0.56; 95% CI 0.10–3.05; *p* = 0.51).

### Revisions, and survivorship

**Figure 3:**
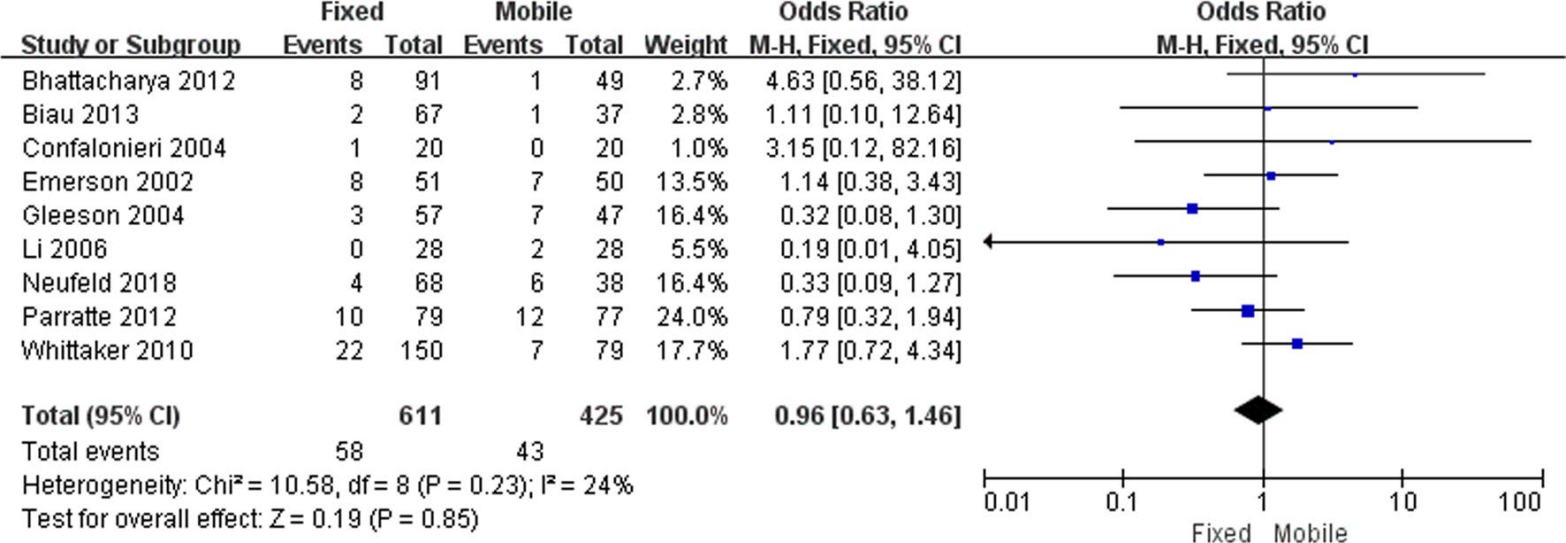
Forest plot for the incidence of revisions following the FB and MB UKAs. Nine studies were analyzed and showed in the forest plot. Since I^2^ = 24% and *p* = 0.23, a fixed effect model was used (RevMan 5, version 5.3, URL: https://training.cochrane.org).

A total of 58 knees from the FB group experienced failure and underwent subsequent revisions, the time of failure ranged from 4-days to 15-years; meanwhile, a total of 43 knees from the MB group underwent subsequent revisions and the time of failure ranged from 6-weeks to 17.7-years. Meta-analysis revealed no significant difference in the incidence of revisions between the two groups (OR = 0.96; 95% CI 0.63–1.46; *p* = 0.85) (Fig. 3).

In the FB group, complications consisted of progression of arthritis, polyethylene wear, aseptic loosening, and persistent pain. In the MB group, complications consisted of progression of arthritis, aseptic loosening, bearing dislocation, and persistent pain. No significant differences were found in the incidence for progression of arthritis (*p* = 0.33), aseptic loosening (*p* = 0.45), persistent pain (*p* = 0.84), and overall reoperation (*p* = 0.32) between the two groups. However, the differences in frequency of polyethylene wear (*p* = 0.02) and bearing dislocation (*p* = 0.03) were statistically significant between the two groups. In addition, the failure time for bearing dislocations was 0.6-years in the MB group and it was 8.3-years for polyethylene wear in the FB group after surgery. Additional details about complications are shown in Table 2.

**Table 2:** The clinical complications for FB and MB prostheses.

Complications	Study number	FB		MB		OR (95% CI)	*p* value
		Revisions (knees)	Mean failure time	Revisions (knees)	Mean failure time		
Progression of arthritis	5	16 (439)	7.2 y (25 m–15 y)	15 (293)	7.0 y (1.4–11 y)	0.70 [0.34, 1.44]	0.33
Aseptic loosening	7	9 (524)	6.4 y (25 m–14.6 y)	9 (368)	6.0 y (1–17.7 y)	0.70 [0.27, 1.77]	0.45
Persistent pain	6	7 (437)	2.9 y (1.1–7.1 y)	4 (283)	5.3 y (2.8–8.4 y)	1.14 [0.33, 3.91]	0.84
polyethylene wear	3	15 (280)	8.3 y (2–10 y)	1 (206)	3 y	11.60 [1.52, 88.57]	0.02
Bearing dislocation	5	0 (404)	NA	8 (290)	0.6 y (2 m–2 y)	0.04 [0.00, 0.71]	0.03

*FB* fixed-bearing, *MB* mobile-bearing, *y* years,* m* months, *N* none, *NA* not applicable.

Four studies demonstrated that survivorship at 5-years, 10-year, and 20-years were 95.6%, 90.9% and 83% in the FB group and 97%, 92% and 80% in the MB group respectively, with a mean follow-up ranging from 3.6 to 17.2-years (Table 3)^1,10,16,18^. Cumulatively these studies suggest similar implant survivorship between the two groups (OR = 1.38; 95% CI 0.83–2.30; *p* = 0.21). However, using the Cox regression analysis model, Bhattacharya et al.^13^ demonstrated better survivorship in the MB group (*p* < 0.05).

**Table 3:** Results of survivorship in unicompartmental knee arthroplasty.

Author	Technique	Number of knees (patients)	Survivorship	Mean follow-up duration	*p* value
Bhattacharya^13^	FB	91 (79)	8.8% at 74 months*	44.7 months	< 0.05
	MB	49 (44)	2.0% at 119 months*	67.6 months	
Emerson^1^	FB	51 (45)	95% at 6 years; 92% at 11 years	7.7 years	NA
	MB	50 (43)	97% at 6 years; 92% at 11 years	6.8 years	
Neufeld^21^	FB	68 (56)	90.9% at 10 years	11.5 years	0.102
	MB	38 (33)	82.9% at 10 years	14.2 years	
Parratte^10^	FB	79 (75)	83% at 20 years	17.2 years	NA
	MB	77 (72)	80% at 20 years		
Whittaker^16^	FB	150 (117)	95.6% at 5 years	8.1 years	NA
	MB	79 (62)	89.3% at 5 years	3.6 years	

*FB* fixed-bearing, *MB* mobile-bearing, *NA* not applicable.

*Revision rate.

### Publication bias

**Figure 4:**
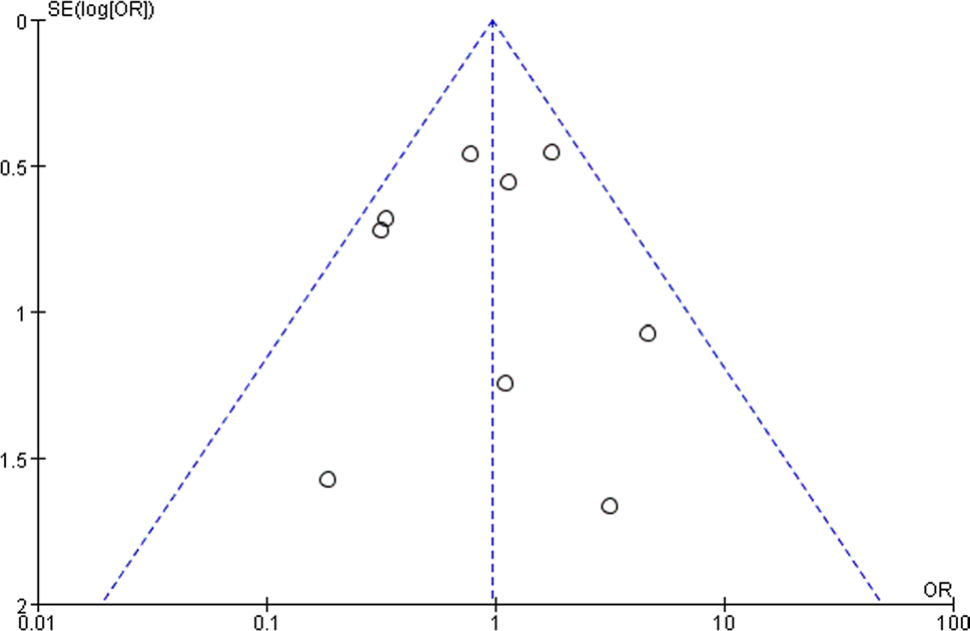
Funnel plot demonstrating minimal publication bias from revision outcome. Nine studies were analyzed, the abscissa axis showed the odds ratio, and the vertical axis showed the standard error (STATA, version 12.0, URL: https://www.stata.com).

The publication bias was assessed using a funnel plot, which demonstrates the relationship between the size of the study sample and the accuracy of the estimated treatment effect. The assessment of publication bias using the incidence of revisions indicated no substantial evidence of bias (*p* = 0.893) (Fig. 4).

## Discussion

UKA comes in two basic prosthesis designs, FB and MB, with disagreement as to which design has superior results^5,8,9^. We have conducted a meta-analysis of all available studies comparing FB to MB UKA prostheses.

Only comparative studies were included in the current meta-analysis. While many studies showed favorable results with fixed or mobile bearing design, they only reported results of a single implant and were therefore excluded from this study^24,25^. The most important finding from our study is that FB and MB prostheses did not differ in terms of: (1) clinical outcomes; (2) radiographic outcomes; (3) revision rates, or (4) survivorship. However, the differences in modes and timing of failures were evident between the two prostheses, bearing dislocation led to earlier failures in the MB prosthesis, while later failures were related to polyethylene wear in the FB prosthesis group.

Differences in clinical outcomes between FB and MB prostheses has been reviewed with each study using different functional measurement indexes. Some studies^15,19^ found better results in knee functional scores with the MB group, while Inoue et al.^12^ and Ozcan et al.^11^ found higher postoperative KSS in the FB group. Interestingly, Peersman et al.^24^ demonstrated that there was a tendency for the KSS to deteriorate more rapidly in MB than FB prostheses between 10 and 15 years. However, none of these studies demonstrated significantly better results for one prosthesis over the other. No significant statistical differences were found between the FB and MB groups on all knee scores measured in this study (Fig. 2).

RLL have previously been reported at almost quintuple the rate in FB UKA’s compared to MB prostheses^20^, while other studies^10,18^ found the opposite results. In this meta-analysis, the incidence of RLL was higher in the MB group compared to the FB group (42.0% MB vs. 19.0% FB) based on limited sample sizes, no significant difference was found (*p* = 0.75). In addition, recent studies suggest that the presence of RLLs is irrelevant to knee scores and revision rates in mid- and long-term follow-up^10,16,26,27^. Therefore, longer-term follow-up RCTs are needed to find the true relationship between the incidence of radiolucent lines and survivorship between different prostheses.

A previous meta-analysis^28^ suggested that the major complications which required reoperation following UKAs consisted of aseptic loosening, progression of arthritis and wear of the polyethylene insert. Some in vitro studies^6,29^ based on knee simulators reported lower wear rates of FB prostheses compared to MB prostheses, while Manson et al.^30^ found that wear was a complication inherent to the design of FB prosthesis caused by the fatigue and sheer stress-related mechanism, secondary to higher surface deformation and delamination in comparison to MB prosthesis. In comparison, MB prostheses reduce overall polyethylene wear by increasing the contact area and congruency while minimizing constraints and maintaining normal knee motion^31^. In MB prostheses, wear of the polyethylene is mainly due to the abrasive and adhesive mechanism^28^. Bearing dislocation is a unique complication in MB prostheses. Any under correction of the deformity may play a role in the medial compartment stresses contributing to bearing dislocation. Considering this, surgeons may be inclined to choose a tight knee and risk slight overcorrection into valgus^32^. However, overcorrection to valgus can result in greater contact stress and significant load on the lateral compartment, thus accelerating the progression of arthritis. In contrast, FB prostheses could offload the lateral compartment and thereby slow the progression of arthritis^28^. In the current study, a higher incidence of polyethylene wear was clearly demonstrated in the FB group, and bearing dislocation was only present in MB group (Table 2).

Survivorship differed between prosthesis design in most studies. Whittaker et al.^16^ reported that the 5-year survival, with revision to TKA as the end point, was 88% for the MB implants and 96% for the FB implants. Reporting on 38 MB and 68 FB knees with a mean follow-up of 14.2-years and 11.5-years, Neufeld et al.^18^ demonstrated that the 10-year survivorship of the MB implants was 82.9% and the FB implants was 90.9%. In addition, a comparative study^10^ with a minimum 15-year follow-up reported that 20-year survivorship was 83% in the FB group and 80% in the MB group. Similar to our study, differences of survivorship between the two prostheses in these studies were not significant (Table 3). To assess the true differences in the lifespan of UKA implants, future higher quality RCTs are needed.

Several limitations should be presented in our study. First, we included 14 non-randomized controlled trials (nRCTs) of low level of evidence, which increases the risk of bias and other cofounding variables. Second, publication bias might affect the outcomes, as we did not search for unpublished studies. However, the funnel plot demonstrated minimal evidence of publication bias (Fig. 4). Finally, due to the high proportion of missing data and the observational nature of the included studies, the results should be interpreted with caution. In our study, no significant differences in knee scores were found between the two prostheses, which may be due to the limited studies and incomplete data. Therefore, future larger scale and long-term follow-up RCTs with complete data directly comparing the knee functional scores between FB and MB UKAs are needed. Theoretically, different complications mean different revision surgeries, such as replacing polyethene insert, UKA, Bi-KA or TKA, while most of present studies don’t mention it. Lacking the specific surgery of revision is another disadvantage of our study, we will analyze the tendency of the type of revision surgeries in these two designs of UKA in future study.

In conclusion, both Fixed Bearing and Mobile Bearing prostheses provide excellent knee scores, radiological outcomes, and survivorship in Unicompartmental Knee Arthroplasties. The superiority of one prosthesis over the other could not be demonstrated and each have their merits and indications. Patient selection and proper technique are required to ensure the Mobile Bearing prosthesis can survive beyond the short-term dislocation complication to enable excellent implant longevity.

## Materials and methods

This meta-analysis was reported in accordance with the Preferred Reporting Items for Systematic Reviews and Meta-Analyses (PRISMA) Statement^33^. Relevant studies published prior to April 21, 2020 were identified by searching PubMed, Embase and the Cochrane Library with no language restrictions. The following search terms, including their variations, were used: “Arthroplasty, Replacement, Knee”, “Unicompartmental”, “Unicondylar”, “Fixed”, “Mobile”, “Bearing”, and Boolean operators (AND, OR) for various combinations. After the initial electronic search, relevant articles and their bibliographies were searched manually.

To minimize any possible selection bias, all studies that satisfied the search strategy were reviewed and included if they met the following criteria: (1) studies which compare FB and MB knee prostheses directly; (2) patients with isolated medial compartment arthritis of the knee; and (3) studies which report clinical or radiographic outcomes, revisions, or survivorship. The exclusion criteria included: (1) review articles, letter or comment; (2) studies which only describe either FB or MB knee prostheses; (3) patients with lateral compartment arthritis or patellofemoral arthritis of the knee; (4) also excluded if the results of comparison were not reported or the data could not be extracted from the published results. Two reviewers (WCZ, JPW) independently assessed each of the studies for eligibility for inclusion. All disagreements were resolved through discussion to reach a final consensus.

All information regarding relevant results were recorded. Data of participants included: number of knees/patients, demographic data (age, sex, weight, height, etc.), duration of the follow-up, and type of prosthesis. The outcome measures included knee scores, range of motion (ROM), femoral-tibial angle (FTA), hip-knee-ankle angle (HKA), incidence of radiolucent lines (RLLs), incidence and timing of revisions, and survivorship. The levels of evidence were assessed using a published rating system^34^.

Heterogeneity was determined by estimating the proportion of between-study inconsistencies by examining actual differences between studies identified in the data extraction tables. Heterogeneity is expressed as p and I^2^; if *p* > 0.10 and I^2^ < 50%, a fixed effects model was adopted; otherwise, a random effects model was chosen. Continuous data was assessed using weighted mean difference (WMD) and associated 95% CI (confidence intervals), adopting the Mantel–Haenszel method^35^; study-specific OR (Odds Ratio) and associated 95% CI were used to determine the value of dichotomous data.

Forest plots were used to graphically present the results of individual studies and the respective pooled estimate of effect size. Statistical significance was regarded as a *p* < 0.05. Publication bias was assessed using a funnel plot of the outcome measure recorded in the largest number of clinical trials^36^. We assessed funnel plot asymmetry using Begg and Egger tests and defined significant publication bias as a *p* < 0.1. Review Manager (RevMan, version 5.3) for Windows and the Cochrane collaboration were used to perform all statistical analyses. Publication bias was assessed using STATA (version 12.0).

## Data Availability

The datasets generated during and/or analysed during the current study are available from the corresponding author on reasonable request.

## References

[1] Emerson RH, Hansborough T, Reitman RD, Rosenfeldt W, Higgins LL (2002). Comparison of a mobile with a fixed-bearing unicompartmental knee implant. Clin Orthop. Relat. Res..

[2] Smith TO, Hing CB, Davies L, Donell ST (2009). Fixed versus mobile bearing unicompartmental knee replacement: a meta-analysis. Orthop. Traumatol. Surg. Res..

[3] Bonutti PM, Dethmers DA (2008). Contemporary unicompartmental knee arthroplasty: fixed vs mobile bearing. J. Arthroplasty.

[4] O'Connor JJ, Goodfellow JW (1996). Theory and practice of meniscal knee replacement: designing against wear. Proc. Inst. Mech. Eng. H.

[5] Gleeson RE, Evans R, Ackroyd CE, Webb J, Newman JH (2004). Fixed or mobile bearing unicompartmental knee replacement? A comparative cohort study. Knee.

[6] Brockett CL, Jennings LM, Fisher J (2011). The wear of fixed and mobile bearing unicompartmental knee replacements. Proc. Inst. Mech. Eng. H.

[7] Argenson JN, Parratte S (2006). The unicompartmental knee: design and technical considerations in minimizing wear. Clin. Orthop. Relat. Res..

[8] Catani F (2012). Muscle activity around the knee and gait performance in unicompartmental knee arthroplasty patients: a comparative study on fixed- and mobile-bearing designs. Knee Surg. Sports Traumatol. Arthrosc..

[9] Confalonieri N, Manzotti A, Pullen C (2004). Comparison of a mobile with a fixed tibial bearing unicompartimental knee prosthesis: a prospective randomized trial using a dedicated outcome score. Knee.

[10] Parratte S, Pauly V, Aubaniac JM, Argenson JN (2012). No long-term difference between fixed and mobile medial unicompartmental arthroplasty. Clin. Orthop. Relat. Res..

[11] Ozcan C (2018). Fixed-bearing unicompartmental knee arthroplasty tolerates higher variance in tibial implant rotation than mobile-bearing designs. Arch. Orthop. Trauma Surg..

[12] Inoue A (2016). Comparison of alignment correction angles between fixed-bearing and mobile-bearing UKA. J. Arthroplasty.

[13] Bhattacharya R, Scott CE, Morris HE, Wade F, Nutton RW (2012). Survivorship and patient satisfaction of a fixed bearing unicompartmental knee arthroplasty incorporating an all-polyethylene tibial component. Knee.

[14] Bini S, Khatod M, Cafri G, Chen Y, Paxton EW (2013). Surgeon, implant, and patient variables may explain variability in early revision rates reported for unicompartmental arthroplasty. J. Bone Jt. Surg. Am..

[15] Verdini F (2017). Assessment of patient functional performance in different knee arthroplasty designs during unconstrained squat. Muscles Ligaments Tendons J..

[16] Whittaker JP (2010). Does bearing design influence midterm survivorship of unicompartmental arthroplasty?. Clin. Orthop. Relat. Res..

[17] Biau DJ, Greidanus NV, Garbuz DS, Masri BA (2013). No difference in quality-of-life outcomes after mobile and fixed-bearing medial unicompartmental knee replacement. J. Arthroplasty.

[18] Neufeld ME, Albers A, Greidanus NV, Garbuz DS, Masri BA (2018). A comparison of mobile and fixed-bearing unicompartmental knee arthroplasty at a minimum 10-year follow-up. J. Arthroplasty.

[19] Lu M, Hu G, Li Z, Cao X (2017). LINK fixed-bearing versus Oxford mobile-bearing unicompartmental knee arthroplasty for medial unicompartment knee osteoarthritis. Chin. J. Tissue Eng. Res..

[20] Li MG (2006). Mobile vs fixed bearing unicondylar knee arthroplasty: a randomized study on short term clinical outcomes and knee kinematics. Knee.

[21] Emerson RH, Head WC, Peters PC (1992). Soft-tissue balance and alignment in medial unicompartmental knee arthroplasty. J. Bone Jt. Surg. Br.

[22] Tecame A, Savica R, Rosa MA, Adravanti P (2019). Anterior cruciate ligament reconstruction in association with medial unicompartmental knee replacement: a retrospective study comparing clinical and radiological outcomes of two different implant design. Int. Orthop..

[23] Kennedy WR, White RP (1987). Unicompartmental arthroplasty of the knee Postoperative alignment and its influence on overall results. Clin. Orthop. Relat. Res..

[24] Peersman G, Stuyts B, Vandenlangenbergh T, Cartier P, Fennema P (2015). Fixed- versus mobile-bearing UKA: a systematic review and meta-analysis. Knee Surg. Sports Traumatol. Arthrosc..

[25] Panni AS, Vasso M, Cerciello S, Felici A (2012). Unicompartmental knee replacement provides early clinical and functional improvement stabilizing over time. Knee Surg. Sports Traumatol. Arthrosc..

[26] Mercier N, Wimsey S, Saragaglia D (2010). Long-term clinical results of the Oxford medial unicompartmental knee arthroplasty. Int. Orthop..

[27] Gulati A (2009). The incidence of physiological radiolucency following Oxford unicompartmental knee replacement and its relationship to outcome. J. Bone Jt. Surg. Br..

[28] Ko YB, Gujarathi MR, Oh KJ (2015). Outcome of unicompartmental knee arthroplasty: a systematic review of comparative studies between fixed and mobile bearings focusing on complications. Knee Surg. Relat. Res..

[29] Kretzer JP (2011). Wear analysis of unicondylar mobile bearing and fixed bearing knee systems: a knee simulator study. Acta Biomater..

[30] Manson TT, Kelly NH, Lipman JD, Wright TM, Westrich GH (2010). Unicondylar knee retrieval analysis. J. Arthroplasty.

[31] Kendrick BJ (2010). Polyethylene wear in Oxford unicompartmental knee replacement: a retrieval study of 47 bearings. J. Bone Jt. Surg. Br..

[32] Kozinn SC, Scott R (1989). Unicondylar knee arthroplasty. J. Bone Jt. Surg. Am..

[33] Moher D, Liberati A, Tetzlaff J, Altman DG (2009). Preferred reporting items for systematic reviews and meta-analyses: the PRISMA statement. PLoS Med..

[34] Wright JG, Swiontkowski MF, Heckman JD (2003). Introducing levels of evidence to the journal. J. Bone Jt. Surg. Am..

[35] Mantel N, Haenszel W (1959). Statistical aspects of the analysis of data from retrospective studies of disease. J. Natl. Cancer Inst..

[36] Egger M, Davey Smith G, Schneider M, Minder C (1997). Bias in meta-analysis detected by a simple, graphical test. BMJ.

